# Plasma atropine concentrations associated with decreased intestinal motility in horses

**DOI:** 10.3389/fvets.2022.951300

**Published:** 2022-09-02

**Authors:** Carl Ekstrand, Peter Michanek, Ronette Gehring, Anna Sundell, Annika Källse, Mikael Hedeland, Lena Ström

**Affiliations:** ^1^Department of Biomedicine and Veterinary Public Health, Division of Pharmacology and Toxicology, Swedish University of Agricultural Sciences, Uppsala, Sweden; ^2^Department of Population Health Sciences, Division of Veterinary and Comparative Pharmacology, Utrecht University, Utrecht, Netherlands; ^3^Department of Medicinal Chemistry, Division of Analytical Pharmaceutical Chemistry, Uppsala University, Uppsala, Sweden; ^4^Department of Clinical Sciences, Division of Large Animal Surgery, Swedish University of Agricultural Sciences, Uppsala, Sweden

**Keywords:** atropine sulfate, colic, equine, pharmacokinetics, pharmacodynamics

## Abstract

**Introduction:**

Atropine is an essential part of the treatment protocol for equine uveitis. Topical atropine administration has been associated with decreased intestinal motility and abdominal pain in horses. Experimental studies have indicated that frequent dosing is associated with a higher risk than dosing every 6 h. Unfortunately, no quantitative pharmacodynamic data for inhibition of the equine gut are published.

**Materials and methods:**

Eight standardbred horses were assigned to receive either atropine or saline (control) to be infused over 30 min in a two-treatment cross-over design. Atropine concentrations in plasma were measured using ultra-high-performance liquid chromatography–tandem mass spectrometry. Intestinal motility was measured using borborygmi frequency and electrointestinography (EIG). Experimental data were analyzed using a non-linear mixed effects model. The model was then used to simulate different dosing regimens.

**Results:**

Atropine significantly decreased borborygmi response and EIG response. Six horses developed clinical signs of abdominal pain. The pharmacokinetic typical values were 0.31, 1.38, 0.69, and 1.95 L/kg·h for the volumes of the central, the highly perfused, the scarcely perfused compartments, and the total body clearance, respectively. The pharmacodynamic typical values were 0.31 μg/L and 0.6 and 207 nV^2^7 cpm for the plasma concentration at 50% of the maximum response and the maximum response and the baseline of cecal EIG response, respectively. Six different dosing regimens of topical atropine sulfate to the eye (0.4 and 1 mg every hour, every 3 h, and every 6 h) were simulated.

**Conclusion:**

The IV PK/PD data coupled with simulations predict that administration of 1 mg of topical atropine sulfate administered to the eye every hour or every 3 h will lead to atropine accumulation in plasma and decreased intestinal myoelectric activity. Administration every 6 h predicted a safe dosing regimen in full-sized horses. Clinical studies would be valuable to confirm the conclusions. For smaller equids and horses put at risk for colic due to othercauses, droplet bottles that deliver 40 μl of 1% atropine sulfate per drop or less may be used to lower the risk further.

## Introduction

Atropine is an alkaloid anti-cholinergic drug acting as a non-selective antagonist at muscarinic receptors (1). It increases heart rate, relaxes smooth muscle cells, and decreases salivation and mucus secretion. Relaxation of smooth muscle cells in the gastrointestinal tract might decrease gastrointestinal motility and impair transport through the intestines. In sensitive species such as horses, clinical signs of abdominal pain is a well-described side effect of atropine administration.

In equine ophthalmology, the main use of atropine is as a topical mydriatic and cycloplegic in treatment protocols for uveitis. Uveitis causes ciliary muscle spasms and pupillary contraction (miosis). The spasm is painful and chronic complications may occur, including synechia between tissues in the eye that can cause persistent pupil constriction, glaucoma, and decreased vision (2). Topical atropine (eye drops) reverse ciliary muscle spasm and the pupil dilates, which relieves pain and decreases the risk for synechia and permanently decreased vision. Different dosing regimens have been reported from experimental studies, with or without side effects. Hourly topical administration of 1 mg atropine sulfate to the eye has been associated with clinical signs of abdominal pain in horses (3). The most likely explanation was systemic absorption of atropine that inhibited intestinal motility. If the drug was administered every 6 h instead, clinical signs of abdominal pain were absent, suggesting a difference in systemic atropine exposure between the two dosing regimens (4). Recently, pharmacokinetic modeling and simulation indicated a short atropine plasma terminal half-life and suggested a complete washout from the circulation between administrations in the 6-h protocol (5). In contrast, simulations predicted accumulation of atropine for the 1 mg per h dosing regimen. This could explain the difference in abdominal pain between study results, but the concentration–response relationship between atropine and intestinal motility remains unclear. This study aimed to quantitatively determine the pharmacodynamics of atropine with regard to its effects on intestinal motility to better estimate the risk for abdominal pain and colic in horses following atropine exposure.

## Materials and methods

### Animals

Eight standardbreds (three geldings and five mares) without known systemic or ophthalmic diseases were included in the study. The horses were 8–18 years and weighed 480–675 kg. During the study, horses were kept in single boxes (their home environment). During washout periods, horses were on pasture during the day time and in single boxes during the nights. Water and hay were available *ad libitum* during experiments. The study was approved by the Animal Ethics Committee, Uppsala, Sweden.

### Experimental design

The study was a blinded, randomized cross-over design including two intravenous (IV) constant rate infusions, one active treatment and one control treatment, administered over 30 min using an infusion pump (Volumat Agilia, Fresenus Kabi AG, Hamburg, Germany). For active treatment, atropine sulfate (Atropin Mylan 0.5 mg/ml, Mylan AB, Stockholm, Sweden) corresponding to the atropine doses 7.5 μg/kg (horses #1–4) and 10 μg/kg (horses #5–8) was diluted in saline (9 mg/ml, Fresenius Kabi, Uppsala, Sweden). Saline was used for the control treatment. A minimum of 3 weeks washout period was applied between treatments.

Before the start of the infusion, horses were exercised by walk at a constant pace during 20–30 min using a horse walker (Pro-walker 18-8, Innovation Sandviken, Sandviken, Sweden) familiar to the horses. One IV catheter (MILA international inc. Florence, KY, United States) was placed in each jugular vein (one for infusion and one for sampling) after desensitization of the skin using a prilocaine + lidocaine cream (EMLA 25 + 25 mg/g, Aspen Nordic, Ballerup, Denmark). To prepare for electrointestinography (EIG), the hair over the right flank and abdomen was clipped and the skin was washed with antiseptic soap. *Via* transabdominal ultrasonography, the cecum and the right dorsal colon were identified. After cleaning the area with alcohol, foam conductive adhesive gel electrodes (MAXENSOR, disposable ECG Electrodes, MediMaxTech UK Ltd., Surrey, UK) were applied. Active and reference electrodes were placed over the cecum and right dorsal colon, respectively. A ground electrode was placed on the ventrolateral abdomen. Impedance was kept below 5 kΩ in all recordings. Responses were amplified, filtered, and stored using a Powerlab system [Powerlab 8/35 and BioAmp FE 235, ADInstruments (Europe) Ltd, Chalgrove, UK]. The EIG frequency was measured within a range of 1.8–12 cycles per min (cpm). Baseline responses were recorded before the start of the infusion. Thereafter, responses were recorded for 5 min during pre-established regular intervals for 10 h after the start of the infusion (see protocol below). EIG responses were analyzed by running spectrum method with fast Fourier transform (FFT), and the total EIG power (nV^2^ · cpm) was evaluated.

Borborygmi frequency was monitored through auscultation for 1 min per quadrant and scored as followed: absent (0), intermittent (1), and continuous (2) over the observation period. The sum of the scores for all four quadrants (total scores) was used in statistical analyses.

### Data collection and blood sampling protocols

Blood was collected using EDTA-coated tubes at time 0 (pre-dose), at 5, 10, 20, 30, 32, 35, 40, 45, 50 min and 1, 1.25, 1.5, 1.75, 2, 2.33, 2.67, 3, 3.5, 4, 5, 6, 8, and 10 h after the start of infusion. The samples were centrifuged at 1,500 *g* for 10 min before plasma was transferred to new tubes and immediately frozen to −20°C. At the end of the day, plasma samples were transferred to −70°C pending analyses. Borborygmi frequency was collected at time 0 (pre-dose), at 0.25, 0.5, 0.75, 1, 1.25, 1.5, 1.75, 2, 2.25, 2.5, 2.75, 3, 3.25, 3.5, 3.75, 4, 4.5, 5, 6, 7, 8, 9, and 10 h. The EIG data were collected at 0, 0.08, and 0.33 h and then followed the same protocol as above from 0.5 h.

Horses were constantly monitored throughout the 10-h observation period for behaviors associated with acute abdominal pain, namely, depression, flank watching, weight shifting, restlessness, kicking abdomen, pawing, stretching, sternal recumbency, lateral recumbency, attempt to lie down, and collapse (6).

### Analytical method

Atropine concentration in plasma was quantified at the National Veterinary Institute (SVA) in Uppsala, Sweden, using ultra–high-performance liquid chromatography–tandem mass spectrometry (UHPLC-MS/MS). The system was composed of an Acquity UHPLC coupled to a TQS Micro tandem quadrupole mass spectrometer with an electrospray interface operating in the positive mode (Waters Corporation, Milford, MA, United States). The calibration range was 0.05–60 μg/L plasma. The precision (relative standard deviation) was in the range of 2.1–8.3% and the recovery was 95.5–98%. The analytical method is thoroughly described in Ström et al.'s work (5).

### Data analyses

A non-linear mixed effects (NLME) model was used for Pharmacokinetic (PK) and pharmacodynamic (PD) analyses using Monolix 2020R1 (Lixoft, Antony, France). Model evaluation was performed by graphical inspection of diagnostic plots (individual fits, observed data vs. predicted data, weighted residuals vs. time, weighted residuals vs. concentration and the visual predictive check, VPC), parameter precision, and objective function values (OFVs), that is, −2 × log likelihood (−2LL) and Bayesian Information Criteria (BIC). A three-compartment model with intravenous administration and first-order elimination was fitted to the atropine concentration–time data. Atropine acts as an antagonist on muscarinic receptors. Hence, a direct response (sigmoidal *I*_*max*_) model was fitted to both cecum and colon EIG data. The PK model was parameterized using Clearance (*Cl*), the volume of the central compartment (*V*_1_), the highly perfused compartment (*V*_2_), the poorly perfused compartment (*V*_3_), inter-compartmental clearance from compartment *V*_1_ to compartment *V*_2_ (*Q*_1_), and inter-compartmental clearance from compartment *V*_1_ to compartment *V*_3_ (*Q*_2_). The PD model was parameterized by means of four parameters: The baseline of response (*R*_0_), the atropine plasma concentration at 50% of the response (*IC*_50_), maximum inhibition (*I*_*max*_), and a sigmoidal parameter (*n)*, also called Hills coefficient. The *n*-parameter was fixed to 1. All parameters were assumed to be log-normally distributed except for the *I*_*max*_ parameter, which was assumed as logit normally distributed. A multiplicative (proportional) residual error model was used. Observations below a lower limit of quantification (LLOQ) were censored, that is, any concentration between 0 and LLOQ could be predicted by the model.

The statistical model for between-subject variability (BSV) was described by:


(1) $$\begin{array}{c} \theta_{i}=\theta_{tv}•\exp(\eta_{i}) \end{array}$$

where θ_*i*_ is the value of the pharmacokinetic parameters in the *i*th horse, θ_*tv*_ is the typical population value of the parameter, and η_*i*_ is the deviation from the corresponding population value associated with the *i*th horse. The standard deviation of the random effects (ω) reported by Monolix was then transformed to a coefficient of variation (CV%) using Equation (2):


(2) $$\begin{array}{c} CV\%=\sqrt{\text{exp}(\omega^{2})-1}•100 \end{array}$$

Shrinkage of the random effects (eta) toward the means was described as:


(3) $$\begin{array}{r} shrinkage=1-\frac{var\left(\eta_{r}\right)}{\omega^{2}} \end{array}$$

where *var(*η_*r*_*)* is the variance of the random effects. When shrinkage for eta was >30%, the random component was not considered to be robustly estimated.

### Simulation of intestinal response after topical atropine administration as eye drops

The plasma concentration–time course and cecum EIG response–time courses after topical atropine administration were simulated in a population of 500 horses based on the PK/PD parameters from this study using Simulx2020R1 (Lixoft, Antony, France). The fitted PK model was adapted for extravascular administration by adding an absorption compartment. The parameter values for the absorption rate constant (*k*_*a*_, 5.95 h^−1^) and bioavailability (*F*, 0.69) were collected from Ström et al. (5). The simulated atropine doses were 1.67 and 0.67 μg/kg representing 0.1 and 0.04 ml of 1% atropine sulfate solution to a 500 kg horse, respectively. Both dose levels were used to simulate three different dosing protocols over 24 h: every hour, every 3 h, and every 6 h.

### Statistical analyses

Independent of the PK-/PD-modeling approach, the EIG- and auscultation response data were subjected to conventional statistical hypothesis testing by means of a linear mixed-effects model. Categorical fixed effects were time and dose. The horse was used as a random effect. Data were compared between doses for every timepoint using Tukey's test for pair-wise comparisons. An *ad hoc* analysis was performed to compare data after atropine and control administration with the pre-administration data. The repeated measures structure of the data was accounted for with respect to both time and individual. Statistical significance was considered when *p* < 0.05. The analyses were performed using the statistical software JMP pro 16.0.0 (SAS institute inc. Cary, NC, United States).

## Results

### Atropine concentration–time course

**Figure 1:**
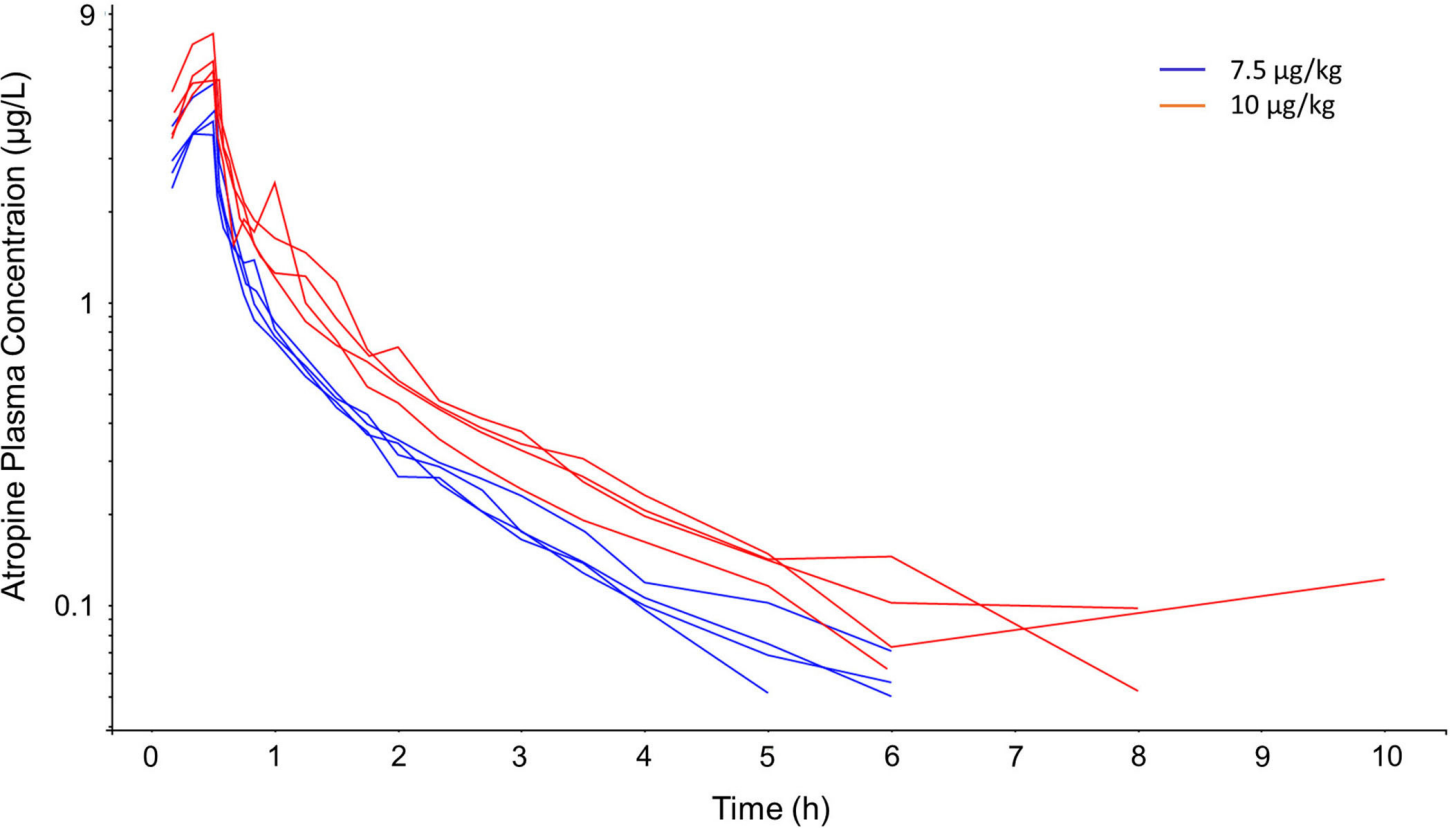
Semi-logarithmic spaghetti plot of observed atropine plasma concentrations over time during and after 7.5 μg/kg (blue lines) and 10 μg/kg (red lines) atropine, administered as a 30 min constant rate infusion to four horses per dosing regimen.

The concentration–time courses were grouped after the two different atropine doses (Figure 1). After dose normalization, data from the two dosing regimens were superimposed. Immediately after the infusion, there was a rapid fall in plasma concentration followed by an intermediate phase and a terminal phase of decreasing concentrations. At 10 h, atropine plasma concentration was quantifiable in only one horse (horse #8). Atropine plasma concentration was below LLOQ (0.05 μg/L) at 8 h in this horse.

**Figure 2:**
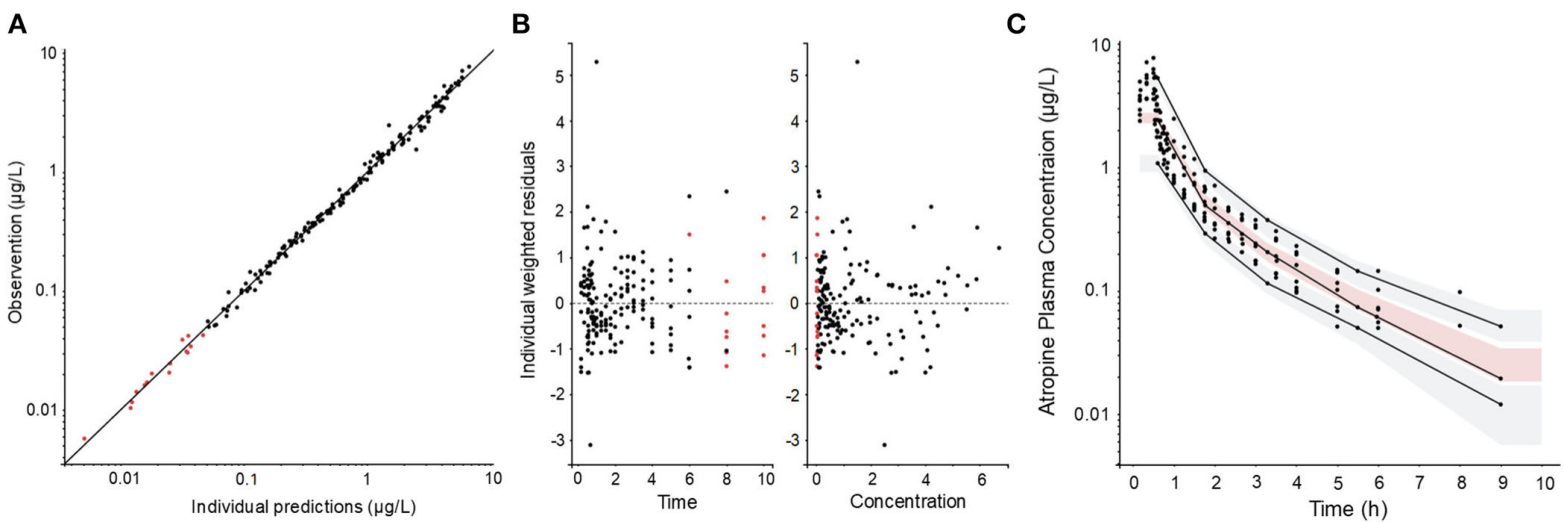
Diagnostic plots of the pharmacokinetic model: **(A)** observations vs. prediction plot, **(B)** individual weighted residuals vs. time and vs. observed concentration, and **(C)** visual predictive check (VPC) **(C)**. Filled black circles represent observed data and filled red circles represent model predicted concentrations below the quantification limit. The solid line in **(A)** represents the line of unity (observation = prediction). The solid lines in **(C)** represent the 10th, 50th, and 90th empirical percentile, respectively. The gray shaded areas in **(C)** are the 10th and the 90th prediction intervals and the red shaded area is the median prediction interval.

The pharmacokinetic three-compartment model fits well into the observed data. The OFVs were −247 and −220 for the three-compartment model compared with −127 and −105 for the two-compartment model for −2LL and BIC, respectively. The pharmacokinetic parameters were estimated with good precision [the relative standard errors (RSE) were below 20%]. The observations vs. predictions were randomly scattered around the line of unity, the vast majority of the weighted residuals were scattered between −2 and 2, and the VPC suggests that the model prediction intervals superimpose the observed data (Figure 2). Shrinkages were below 15% for all PK parameters. The PK model parameters, their RSE, and BSV are given in Table 1.

**Table 1:** Pharmacokinetic model parameter estimates and secondary parameter estimates after 30 min constant rate infusion of atropine in eight horses.

**Model parameters**	**Unit**	**Typical value**	**R.S.E**.	**BSV (%)**
*V*_1_	L/kg	0.31	19.3	9.4
*V*_2_	L/kg	1.38	9.91	24.3
*V*_3_	L/kg	0.69	13.4	20.2
*Cl*	L/kg·h	1.95	4.35	11.0
*Q*_1_	L/kg·h	0.69	14.4	21.2
*Q*_2_	L/kg·h	2.15	17.0	36.1
Residual error parameter		0.12	6.93	

*V*_1_, *V*_2_, *V*_3_*, Cl, Q*_1_, and Q_2_ are the volumes of the central, the highly perfused, and the scarcely perfused compartments, the total body clearance, and the inter-compartmental distribution clearance between *V*_1_ and *V*_2_ and *V*_1_ and *V*_3_, respectively. R.S.E. is the relative standard error of the typical value and BSV (%) is the between-subject variation.

### Intestinal motility

**Figure 3:**
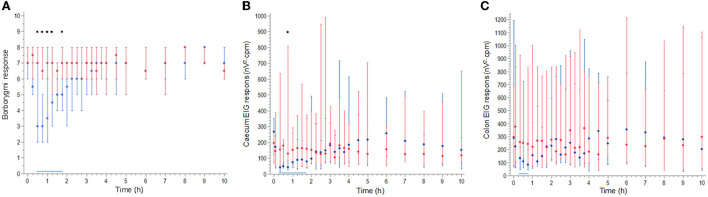
Median (symbols) and range (error bars) borborygmi response **(A)**, cecum EIG response **(B)**, and colon EIG response **(C)** during and after atropine (blue) and control (red) administered as a 30 min constant rate infusion to eight horses. Black stars indicate a significant lower response after atropine than after control treatment. A solid horizontal blue line indicates the time when the response was significantly lower after atropine treatment than at 0 h.

During control treatment, the borborygmi response (total score summarized for all four quadrants) was constantly >5 in all horses (Figure 3). Intermittent or constant borborygmi were present in all quadrants at all observations. There was a significant effect of time (*p* < 0.0001), dose (*p* < 0.0001), and the interaction atropine dose and time (*p* < 0.001). Compared with control treatment, borborygmi response was significantly lower after atropine administration between 0.5 and 1.25 h (*p* < 0.0001) and at 1.75 h (*p* = 0.03). Compared with pre-administration data, atropine decreased borborygmi response at 0.5, 0.75, 1, 1,25 h (*p* < 0.0001), 1.5 h (*p* = 0.004), and 1.75 h (*p* = 0.02). Control treatment did not decrease borborygmi response at any timepoint compared with pre-administration data.

There was a significant effect for the interaction of atropine treatment and time for both cecum EIG response (*p* < 0.0001) and colon EIG response (*p* < 0.0001). Compared with control treatment, cecum EIG response was significantly lower after atropine administration at 0.75 h (*p* = 0.027) (Figure 3). Compared with pre-administration, atropine decreased cecum EIG response at 0.33, 0.5, 0.75 (*p* < 0.0001), 1 h (*p* = 0.004), 1.25 h (*p* = 0.002), 1.5 h (*p* = 0.03), and 1.75 h (*p* = 0.02). Compared with pre-administration, atropine decreased colon EIG response at 0.33 h (*p* = 0.002), 0.5 h (*p* = 0.02), and 0.75 h (*p* < 0.001) (Figure 3). Control treatment did not decrease cecum or colon EIG response compared with pre-administration observations.

**Figure 4:**
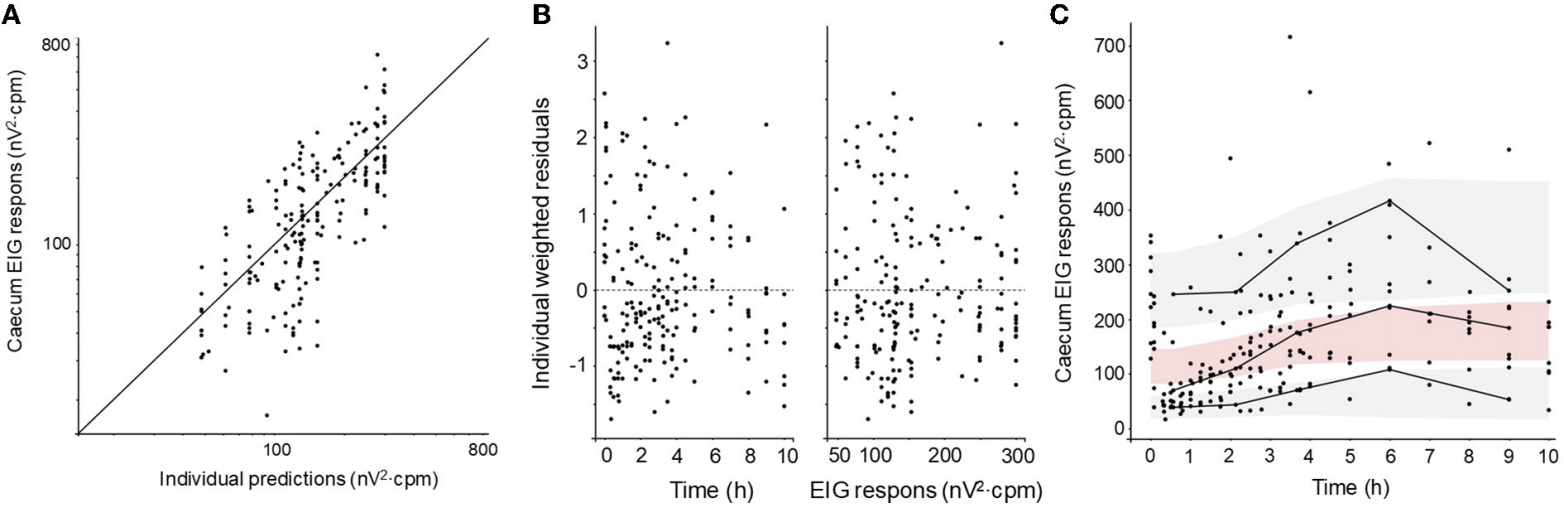
Diagnostic plots of the cecum EIG response pharmacodynamic model: **(A)** observations vs. predictions plot, **(B)** weighted residuals vs. time and vs. observed concentration, and **(C)** visual predictive check (VPC). Filled black circles represent observed data. The solid line in **(A)** represents the line of unity (observation = prediction). The solid lines in **(C)** represent the 10th, 50th, and 90th empirical percentile, respectively. The gray shaded areas in **(C)** are the 10th and the 90th prediction intervals and the red shaded area is the median prediction interval.

**Figure 5:**
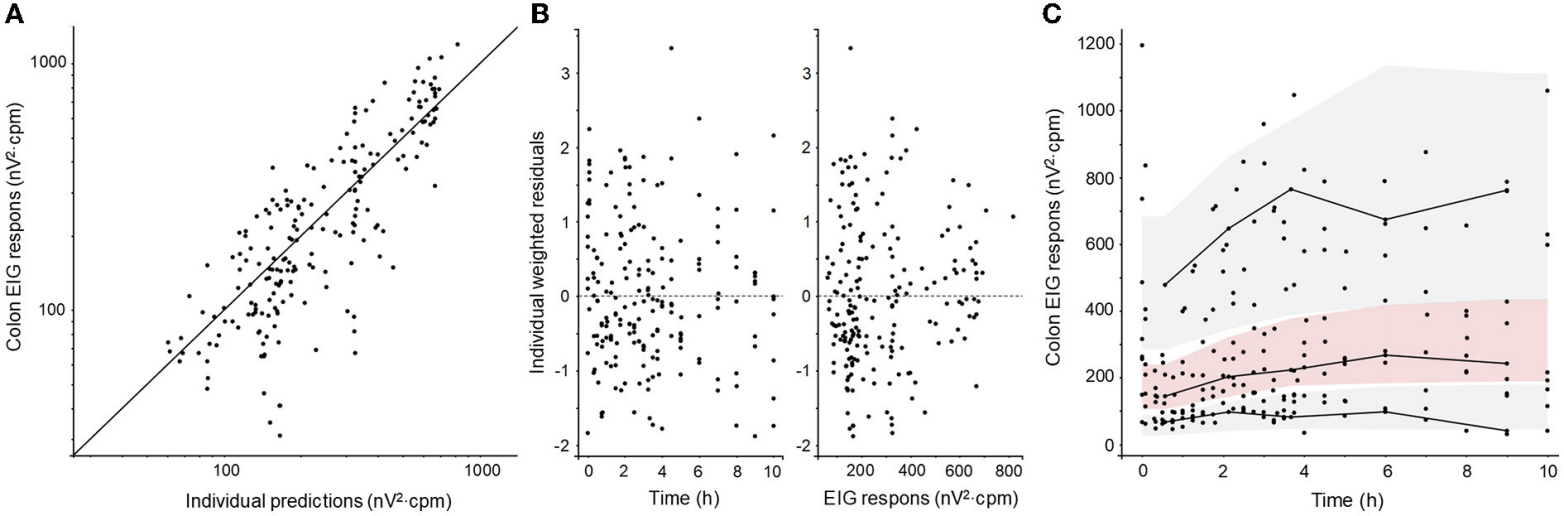
Diagnostic plots of the colon EIG response pharmacodynamic model: **(A)** observations vs. predictions plot, **(B)** weighted residuals vs. time and vs. observed concentration, and **(C)** visual predictive check (VPC). Filled black circles represent observed data. The solid line in **(A)** represents the line of unity (observation = prediction). The solid lines in **(C)** represent the 10th, 50th, and 90th empirical percentile, respectively. The gray shaded areas in **(C)** are the 10th and the 90th prediction intervals and the red shaded area is the median prediction interval.

The PD model was fitted to both cecum and colon EIG response data without major bias (Figures 4, 5). The PD parameters for cecum EIG response were estimated with good precision (RSE below 30%). For colon EIG response, the potency value (*IC*_50_-value) was imprecise (RSE 125%). Other parameters were estimated with acceptable precision. Shrinkages were below 10% for all PD parameters. Pharmacodynamic parameters, their RSE, and BSV are given in Table 2.

**Table 2:** Pharmacodynamic model parameters for inhibition of intestinal motility induced by atropine exposure in horses.

**Model parameters**	**Unit**	**Cecum**			**Colon**		
		**Value**	**R.S.E**.	**BSV(%)**	**Value**	**R.S.E**.	**BSV(%)**
*IC*_50_	μg/L	0.31	26.9	67.1	0.45	125	17.1
*I_*max*_*		0.6	5.44	15.1	0.67	25.0	37.2
*R*_0_	nV^2^ · cpm	207	11.6	29.6	335	39.1	61.0
Residual error parameter		0.48	6.42	–	0.43	8.51	–

IC_50_, *I*_*max*_, *andR*_0_ are the plasma concentrations at 50% of the maximum response, the maximum response (indicating the fractional reduction of the response), and the baseline of response, respectively. R.S.E. is the relative standard error of the typical value and BSV(%) is the between-subject variation.

### Clinical signs of acute abdominal pain

Atropine infusion induced behaviors associated with abdominal pain in six horses (75%): two horses treated with 7.5 μg/kg atropine and four horses treated with 10 μg/kg atropine. Behavior onsets were between 15 and 52 min into the experiment and behavior durations were between 0.29 and 75 min. The behaviors observed were depression, flank watching, and weight shifting.

### Simulation of atropine concentration–time courses and EIG response–time courses

**Figure 6:**
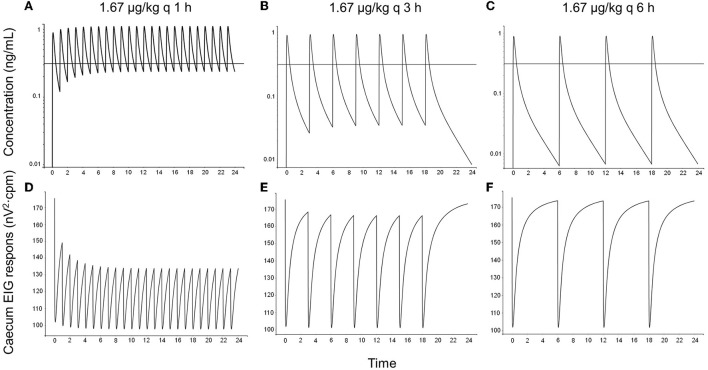
One simulated example of atropine concentration–time courses **(A–C)** and cecum EIG response–time courses **(D–F)** in horses following topical administration of 1.67 μg/kg atropine sulfate as eye drops every hour (left column), every 3 h (middle column), and every 6 h (right column). The dose 1.67 μg/kg represents 100 μl 1% atropine sulfate (corresponding to 835 μg atropine) for a 500 kg horse. The solid black horizontal line in concentration–time plots represent the population value (*IC*_50_-value) for the concentration at 50% of maximal response (0.31 μg/L).

**Figure 7:**
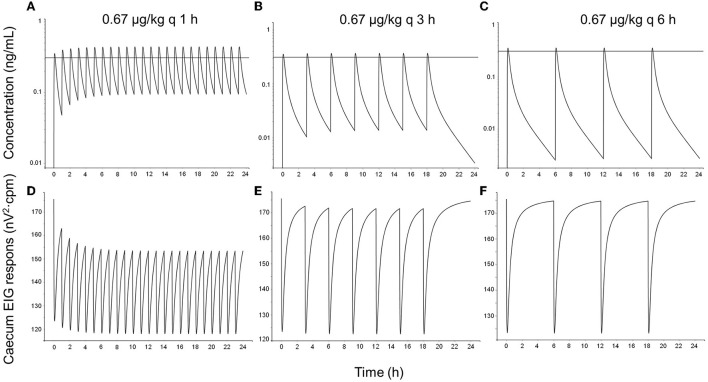
One simulated example of atropine concentration–time courses **(A–C)** and cecum EIG response–time courses **(D–F)** in horses following topical administration of 0.67 μg/kg atropine sulfate as eye drops every hour (left column), every 3 h (middle column), and every 6 h (right column). The dose 0.67 μg/kg represents 40 μl (the average droplet volume delivered by a droplet bottle) 1% atropine sulfate (corresponding to 334 μg atropine) for a 500 kg horse. The solid black horizontal line in concentration–time plots represents the population value (*IC*_50_-value) for the concentration at 50% of maximal response (0.31 μg/L).

Simulations showed that atropine accumulated in plasma after dosing every hour and every 3 h but not after dosing every 6 h (Figures 6, 7). The EIG responses were inhibited in a concentration-related fashion. The dosing regimen 1.67 μg/kg hourly induced the greatest suppression of intestinal myoelectrical activity, both compared with less-frequent dosing and with the lower dose (0.67 μg/kg).

## Discussion

This study is the first quantitative PK/PD study investigating the relationship between dose, atropine concentrations, and intestinal response. The resultant PK/PD model was applied to predict the risk for adverse gastrointestinal side effects given different dosing regimens by means of simulations. This provides a valuable tool for clinicians and veterinary pharmacologists to improve the safety and efficacy of atropine in horses.

The plasma concentrations increased in direct proportion to the dose. This indicates linear PK within the studied concentration range. The use of a three-compartment PK model described the experimental atropine data well. The goodness-of-fit plots presented in Figure 2 show neither bias in the structural model nor the error model. The observed and model predicted concentrations were randomly scattered around the line of unity, the residuals randomly scattered around zero with the majority of residuals between −2 and 2, and the prediction intervals overlapped the empirical percentiles. The typical values for clearance and volume at a steady state (i.e., the sum of the volumes for the respective compartments) were 1.95 L/kg·h and 2.38 L/kg, respectively. This is similar to 1.9 L/kg·h and 1.7 L/kg previously reported using a two-compartment model (5). This was not surprising since both studies were performed using standardbred horses, atropine concentrations were determined using UHPLC/MS-MS, and data were analyzed using NLME.

Atropine administration significantly decreased both borborygmi response and EIG response. Moreover, 75% of the horses developed clinical signs of abdominal pain. Atropine, *in vitro* and *in vivo*, has shown to decrease intestinal motility, increase gastrointestinal transit time, and induce clinical signs of abdominal pain in horses (3, 7–11). Borborygmi frequency has commonly been used to evaluate the equine abdomen both clinically and in experimental pharmacological studies (12–17). Borborygmi response decreased after atropine administration compared with both control treatment and 0 h, similar to what has been described in several previous studies (3, 5, 8, 10). The measurement of borborygmi response is subjective. Previous studies have shown that repeated measurements by the same observer tend to be consistent, but that inter-observer variability is higher (18). Therefore, this experiment was blinded and the same researcher performed all auscultations. However, atropine administration induced pupil dilation. This could have compromised the blinding of the observer. Some subjectivity in the results can therefore not be excluded.

Electrointestinography data, a more objective measurement, were also recorded and used in PK/PD modeling. Percutaneous recording of intestinal myoelectric activity has been suggested to be clinically applicable and a useful tool to evaluate intestinal motility experimentally (19–21). Baseline EIG data showed variability between individuals with a BSV of 30% for the cecum and 60% for the colon (Figure 3). Intestinal motility and emergence of abdominal pain (colic) also vary depending on management, for example, feeding and housing (22–24). Horses in this study were walked before the start of each experimental leg and fed hay during the experiment, both of which increase intestinal motility. The variability together with the conservative statistical model to avoid type I errors are the most probable reasons that only cecum EIG response at 0.75 h was significantly lower than the control treatment. However, the EIG response was also significantly lower after atropine administration compared with pre-administration data.

The pharmacodynamic model was able to quantify the inhibition of intestinal electrical activity induced by atropine exposure following IV administration. The goodness-of-fit plots presented in Figures 4, 5 also suggest a model fit with no bias of either the structural model or the error model. The typical values for the potency (*IC*_50_ values) were 0.31 and 0.45 μg/L for the cecum and the colon, respectively. Cecum EIG response was also significantly lower for a longer period after atropine administration than colon EIG response. This suggests that the cecum is more sensitive to atropine exposure than the colon in fed horses. This is similar to what has previously been shown in fasted horses and after sedation with xylazine (25). No atropine potency values or efficacy values have previously been published in horses.

In humans, the PK/PD relationship for atropine was characterized using heart rate and saliva flow as markers for the response (26, 27). The potency values for heart rate and saliva flow were then estimated to be 6.2 and 3.7 μg/L. This is approximately 10- to 20-fold higher than the *IC*_50_ values presented in the present study, that is, the concentration to achieve half maximum EIG response is lower than that needed for cardiovascular or secretory effects in man. This was unexpected. Larger doses are generally required for inhibition of intestinal motility than for decreasing salivary secretion or vagal tone in other species (28).

In previous studies which investigated the association between ophthalmic atropine treatment and systemic effects, 100 μl of 1% atropine sulfate (corresponding to 835 μg atropine) was administered topically (3–5). Labeled ophthalmic solutions are generally available in dropper bottles that deliver lower volumes, with an average droplet volume of 40 μl (range 25–70 μl) (29). Hence, two doses were simulated in this study; a full dose, 1.67 μg/kg (835 μg/500 kg) and a 40% dose (0.67 μg/kg).

When topical dosing every hour was simulated using the PK/PD data derived after IV dosing in this study combined with literature data from Ström et al. (5), atropine was predicted to accumulate in plasma at concentrations above the *IC*_50_-value for cecum EIG response (0.31 μg/L). Consequently, the intestinal myoelectric activity was predicted to decrease, which would explain why horses developed colic with this dosing regimen (3).

Also with simulated topical dosing, every 3 h atropine concentrations were predicted to peak above 0.31 μg/L, and there was an accumulation of atropine in plasma. However, the short half-life resulted in trough concentrations below the *IC*_50_-value, and inhibition of intestinal myoelectric activity was less than compared with hourly dosing. Ström et al. (5) reported similar results; atropine accumulated in plasma and borborygmi response was lowered after repeated topical administration of 1 mg atropine sulfate. These horses did not show any clinical signs of abdominal pain.

If the 6-h protocol was simulated, no clinically important drug accumulation was predicted. Atropine was predicted to peak above 0.31 μg/L, but the decrease in intestinal myoelectrical activity was of short duration, and the intestinal function over the dosing interval is unlikely to be affected. Consistent with these results, borborygmi response remained unchanged in other experimental studies using the 6 h dosing regimen (4, 5). This dosing regimen is consistent with administration every 4–24 h, which is one of the current dose recommendations for atropine in the treatment of equine uveitis (4, 30).

If a droplet bottle delivering 40 μl per drop is used, the risk for colic decreases further. The lower dose is unlikely to cause colic using the 3- or 6-h dosing interval, based on the simulations performed in this study using IV and literature data. With more frequent dosing, however, the accumulation of atropine in plasma will decrease intestinal motility and might induce clinical signs of abdominal pain during chronic administration. Caution is also advised when horses and ponies smaller than 500 kg are treated since the dose per kg, and consequently, the plasma concentrations, will increase with decreasing weight.

Horses with uveitis treated with atropine might be at risk for colic despite the dosing regimen. In a retrospective study on 337 equids, a univariate analyses suggested that topical use of atropine was associated with a higher risk for colic (31). However, when age and hospitalization time were added to the analysis, they became significant predictors of colic risk, and atropine lost its significance. Other factors that are associated with risk for colic are pain, activity level, change in feed, and stabling conditions (13, 22, 23). Accordingly, horses exposed to any of those factors may be more sensitive to plasma atropine exposure, that is, the potency value for the decrease in intestinal myoelectrical activity might be lower than in healthy horses.

Atropine dilates the pupil and, therefore, exposure to sunlight should be avoided. Also, uveitis many times induces photophobia and pain. Therefore, horses on atropine treatment due to uveitis are often stabled and their activity levels are decreased. Hospitalized horses might also have a change in their diet. These factors are more likely to cause colic than a low daily dose of topical atropine (e.g., 1 mg atropine sulfate every 6 h or less) is used on horses. Moreover, atropine reverses the painful ciliary muscle spasm that might decrease the risk of colic.

In conclusion, the IV PK/PD data coupled with simulations presented here predict that topical administration of 1 mg atropine sulfate every hour or every 3 h leads to drug accumulation and decreased intestinal myoelectric activity. However, topical administration every 6 h was predicted to be a safe option in full-sized horses. Clinical studies would be valuable to confirm these conclusions. For small horses, ponies, and horses put at risk for colic due to other causes (e.g., hospitalization, decreased exercise, environmental stress, pain), droplet bottles that deliver 40 μl 1% atropine sulfate per drop or less may be used to lower the risk for colic further.

## Data availability statement

The raw data supporting the conclusions of this article will be made available by the authors, without undue reservation.

## Ethics statement

The animal study was reviewed and approved by the Regional Animal Ethics Committee, Uppsala, Sweden.

## Author contributions

CE and LS planned the experiment and performed the experiment together with AK, AS, and PM. The data were then analyzed by CE, PM, RG, and MH. CE drafted the manuscript. All authors revised the manuscript and approved the final version of the manuscript.

## Funding

This study was funded by the Petra Lundberg Foundation and the Sveland Foundation for Animal Welfare and Health.

## Conflict of interest

The authors declare that the research was conducted in the absence of any commercial or financial relationships that could be construed as a potential conflict of interest. The handling editor JM declared a past collaboration with the author RG.

## Publisher's note

All claims expressed in this article are solely those of the authors and do not necessarily represent those of their affiliated organizations, or those of the publisher, the editors and the reviewers. Any product that may be evaluated in this article, or claim that may be made by its manufacturer, is not guaranteed or endorsed by the publisher.
